# Non-destructive real-time monitoring of underground root development with distributed fiber optic sensing

**DOI:** 10.1186/s13007-024-01160-z

**Published:** 2024-02-29

**Authors:** Mika Tei, Fumiyuki Soma, Ettore Barbieri, Yusaku Uga, Yosuke Kawahito

**Affiliations:** 1 Institute of Crop Science, National Agriculture and Food Research Organization, 2-1-2 Kannondai, Tsukuba, Ibaraki 305-8518 Japan; 2 Research Institute for Value-Added-Information Generation, Japan Agency for Marine-Earth Science and Technology, 3173-25 Showa-machi, Kanazawa-ku, Yokohama, Kanagawa 236-0001 Japan; 3 Advanced Institute for Marine Ecosystem Change, Japan Agency for Marine-Earth Science and Technology, 2-15 Natsushima, Yokosuka, Kanagawa 237-0061 Japan

**Keywords:** Fiber optic sensing, Root system architecture, Underground monitoring, Digital twins

## Abstract

Crop genetic engineering for better root systems can offer practical solutions for food security and carbon sequestration; however, soil layers prevent the direct visualization of plant roots, thus posing a challenge to effective phenotyping. Here, we demonstrate an original device with a distributed fiber-optic sensor for fully automated, real-time monitoring of underground root development. We show that spatially encoding an optical fiber with a flexible and durable polymer film in a spiral pattern can significantly enhance sensor detection. After signal processing, the resulting device can detect the penetration of a submillimeter-diameter object in the soil, indicating more than a magnitude higher spatiotemporal resolution than previously reported with underground monitoring techniques. Additionally, we also developed computational models to visualize the roots of tuber crops and monocotyledons and then applied them to radish and rice to compare the results with those of X-ray computed tomography. The device’s groundbreaking sensitivity and spatiotemporal resolution enable seamless and laborless phenotyping of root systems that are otherwise invisible underground.

**Supplementary Information:**

The online version contains supplementary material available at 10.1186/s13007-024-01160-z.

## Introduction

The root system architecture (RSA) of plants has important implications for food security, climate action, and bio-inspired civil engineering technologies. For food security, the RSA determines crop resilience to environmental stress [1] and nutrient uptake efficiency [2, 3]. Against climate change, roots and root exudates are natural solutions for carbon sequestration [4]. Deep and densely rooted plants are advantageous for a larger capacity and stability of carbon storage [5–7]. In civil engineering, some geotechnical solutions involving soil excavation, penetration, and anchoring were inspired by biological organisms such as plant roots and earthworms [8]. Biological strategies are optimized for energy efficiency by natural selection and thus can provide useful insights for greener engineering solutions. Elucidating underground RSA can offer relevant solutions for today’s global issues; however, layers of opaque soil hinder easy visualization.

Underground roots have been studied either destructively or non-destructively. In destructive methods, root samples are usually excavated by shoveling [9], coring [10], trenching [11], or gravitation [12], then washed and examined. For example, shovelomics combined with laser ablation tomography is used to anatomically characterize nodal roots [13], and backhoe-assisted monoliths combined with RNA-seq are used to reveal the transcriptomic profiles of rice roots [14]. Destructive methods can bring roots under direct vision but are unsuitable for high-throughput and dynamic experiments because of their single-time and laborious measurement. In non-destructive methods, either the soil or the light property is altered to circumvent the light attenuation of the soil, for example by using transparent soil substitutes [15, 16], X-rays [17–19], and MRIs [20]. These methods may enable high-throughput and semi-automated measurements but may not apply to plants in the field.

Several field monitoring methods have been developed, such as acoustic imaging [21], electrical resistivity tomography [22, 23], electrical impedance tomography [23, 24], ground-penetrating radars (GPR) [25–27], and water potential sensor arrays [12]; however, these methods have yet to achieve sufficient spatial resolution and sensitivity required to detect structures as fine as crop roots. The smallest objects detected using these methods were tree roots with diameters of 5 mm for acoustic imaging [21] and 2.5 mm for GPR [25]. The more direct methods of exposing roots to the camera, such as rhizotrons [28, 29], rhizoboxes [30, 31], and Rhizotubes [32] have been used to observe fine roots; however, these methods are limited in the field of view and may affect the natural environment of the roots and interfere with the processes being investigated.

Therefore, current underground visualization techniques lack spatiotemporal resolution, sensitivity, or range for real-time monitoring of RSA.

To achieve the requisite spatiotemporal resolution and range, a distributed fiber optic sensor (FOS) based on Rayleigh scattering is an emerging technology for monitoring physical quantities such as temperature, strain, and vibration [33]. Fiber optics, which are made of transparent amorphous solids, such as glass and polymers, are widely used in telecommunications. Light waves are trapped in the higher-refractive-index core interfaced with the lower-refractive-index cladding. In contrast to telecommunications, which relies on the transmission of light, FOS exploits the scattering caused by heterogeneity in the core material. Because Rayleigh scattering is material-specific and independent of external energy transfer, the longitudinal coordinates of the FOS can be deciphered using optical frequency-domain reflectometry (OFDR) [34]. When the fiber is locally stretched by mechanical or thermal expansion, light reflection is delayed, which manifests as an induced shift in the reflected spectrum. The degree of expansion is linearly related to the spectral shift of the segment corresponding to the reference site [35]. FOS has been applied to study large-scale dynamics such as earthquakes [36], glacial flows [37], and whales [38]. Innovation is required for its application in root monitoring, which operates on a scale many orders of magnitude smaller in force and energy.

The objective of this study was to overcome the difficulties related to the sensitivity and spatial resolution of FOS required for monitoring root development. To do so, we designed and implemented an original device that significantly enhanced the FOS sensitivity and spatial encoding, accompanied by signal processing and visualization software for RSA determination. The completed device and software were validated by real-time root monitoring for radish and rice over weeks and months, and the virtually reconstructed root structures generally agreed with high-resolution X-ray computed tomography (CT) images of the actual roots.

## Results

### Design and characterization of the sensing device

**Fig. 1:**
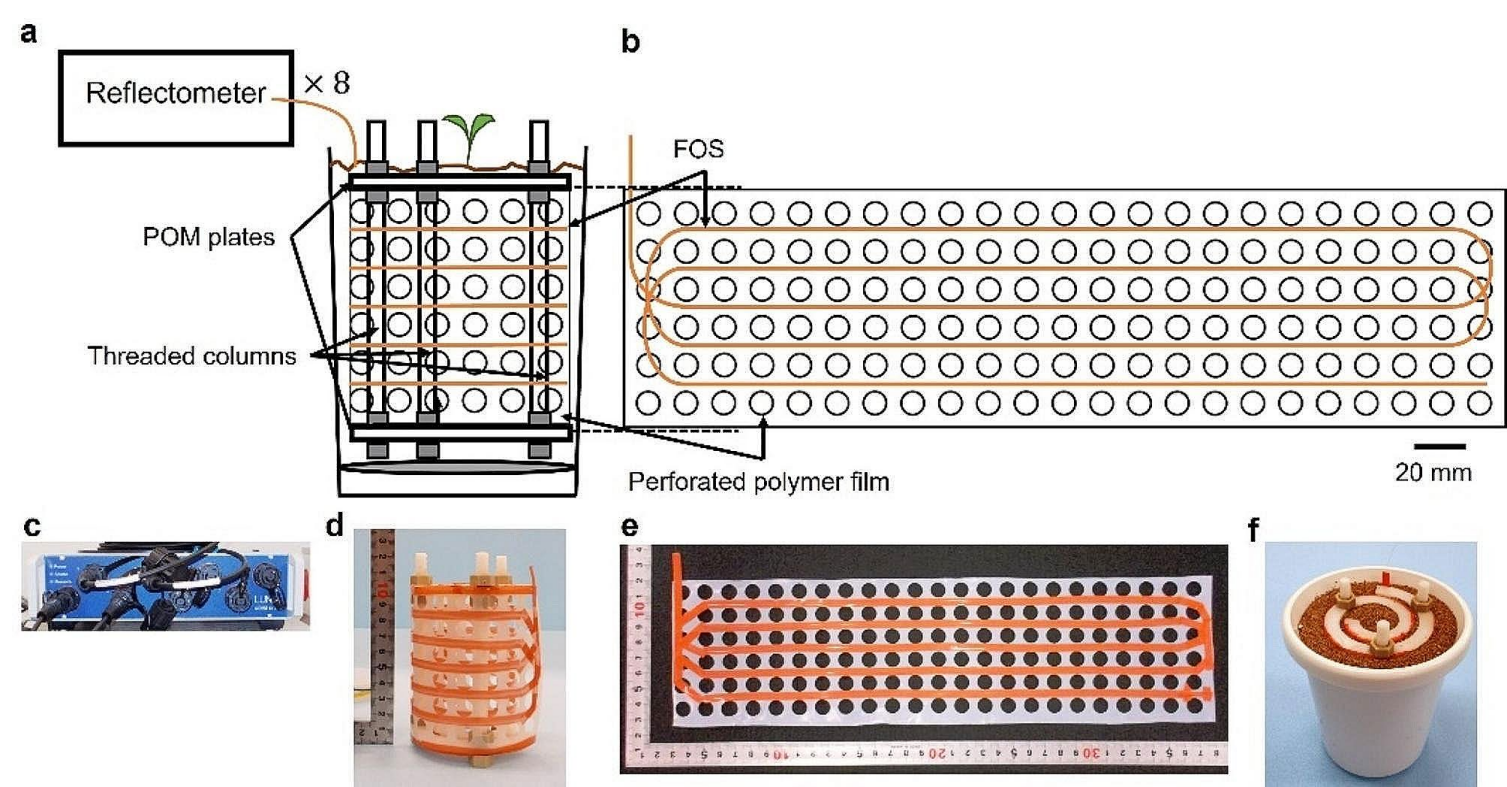
A schematic and photos of a completed FOS device. **(a)** The overall sensing schemes. FOS is attached to a perforated polymer film which is mechanically supported. **(b)** The unfold schematic of the device. **(c)** A photo of the reflectometer. **(d)** The side view of the device. **(e)** The unfold view of the device. **(f)** The device filled with Profile in a cultivation pot

The proposed device is a spatially encoded FOS connected to a commercially available optical reflectometer. Figure 1 shows the complete design of the device. The FOS consisted of a single-mode fiber composed of a GeO_2_-doped silica core, pure silica cladding, and a polyimide coating. To cover the space for the entire root system, the FOS was supported by rigid structures that were extensively and evenly placed in the soil. The combination of structuring and OFDR enabled the spatial encoding of one-dimensional signals. FOS density of 1 per 30 mm was attempted to detect a root diameter of 0.3 mm for 15 µε according to Eq. S1 in Supplementary Theory I.

The device was set in a cultivation pot and then filled with soil-like substrates. The distribution of strains was recorded to evaluate the device’s sensitivity while a thin metal wire, mimicking a root, penetrated at the center. The direct installation of the FOS resulted in a low signal and high noise, as shown in Fig. S1. To enhance the signal gain and stability, the FOS was attached to a perforated polymer film to increase the surface area and plasticity of the sensor. Polyoxymethylene (POM) and polytetrafluoroethylene (PTFE) with Young’s moduli of 3.015 and 0.569 GPa, respectively, were used in the prototype, as shown in Fig. S2. As expected, both polymers improved the gain (stress-to-strain ratio) and stability, with PTFE demonstrating superior performance.

**Fig. 2:**
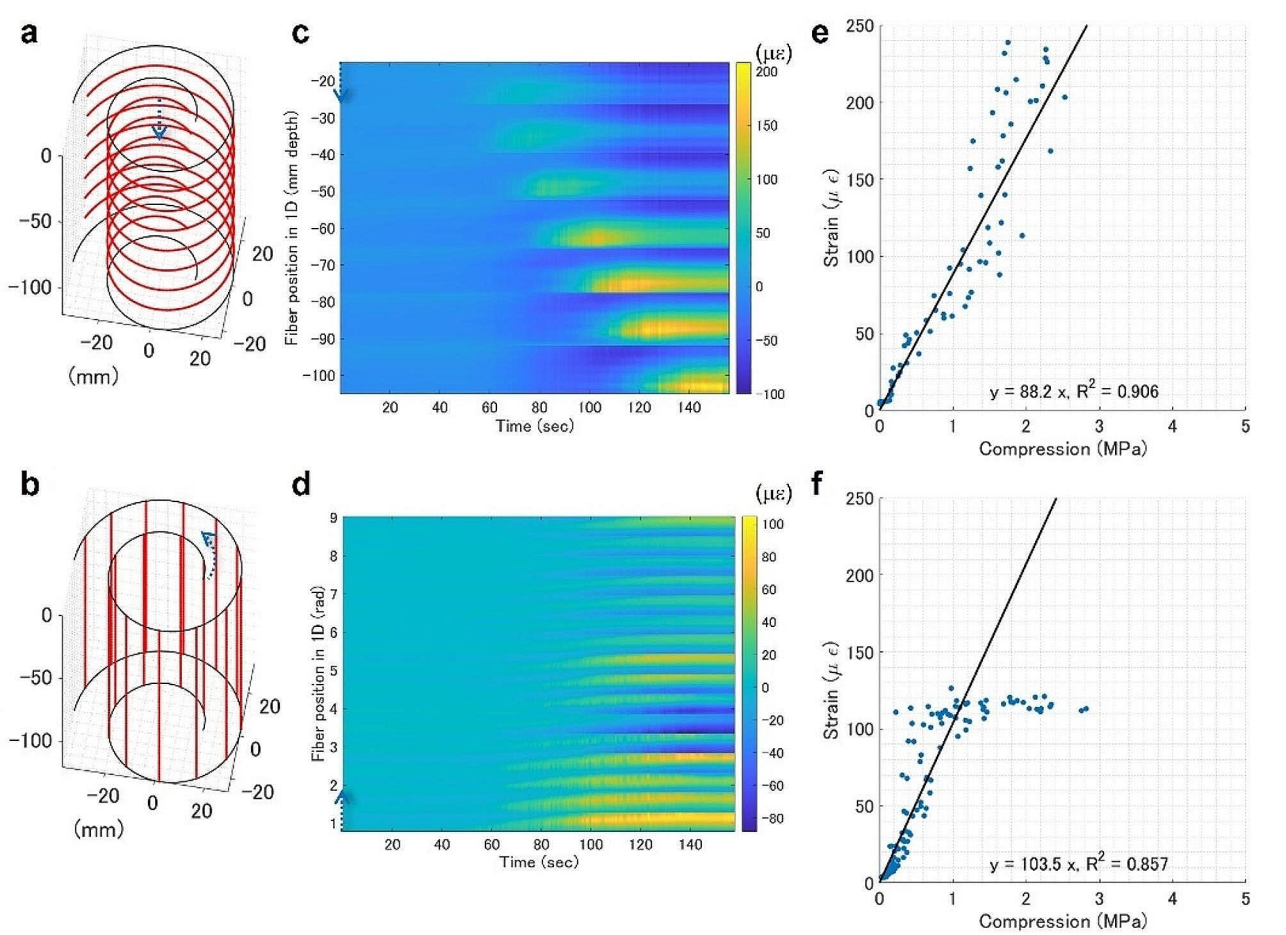
Evaluation of FOS orientation. **(a, b)** FOS placement of a prototype indicated by red lines. **(c, d)** Distributed strain recording from a horizontally or vertically oriented FOS when penetrating 1.2 mm-diameter metal wire at 1 mm/sec into the center of a pot filled with glass beads. **(e, f)** Stress-strain scatter plots of **(c)** and **(d)**, respectively. The fit for linear regression is annotated in the respective panel. For **(f)**, the fit only applies to the linear range (< 1 MPa)

Different FOS orientations were tested on the PTFE films, as shown in Fig. 2. The horizontally and vertically oriented FOS on PTFE exhibited high sensitivity and stability. However, the linearity was lost in the vertical orientation as the strain saturated at 1 MPa compression pressure by the wire. Different penetration sizes and media stiffnesses were tested using agarose gel to fill the cultivation pot with a horizontally oriented FOS on the PTFE film, as shown in Fig. S3. The force required for penetration, as well as the strain, increased as the size of the penetrating object increased or at a higher concentration of agarose. The amplitude of strains was proportional to the force of penetration, with a detection limit of 0.07 N when a metal wire of 0.5 mm diameter penetrated 1.5% agarose gel. This suggests that our device is highly sensitive to the penetration force to the ground when a relatively incompressible material like agarose gel is used as the filler.

Therefore, the complete design of the sensing device consisted of an FOS horizontally fixed on a perforated PTFE film. The intervals between adjacent segments of the FOS were in the range of 15–30 mm. The size and frequency of the perforations were adjusted to reduce interference on root development in a trade-off with signal sensitivity.

### Noise reduction and temperature separation

The FOS was sealed between chemically stable PTFE films within the device to avoid non-target chemical-based effects on the optical signals.

Digital signal processing was also used to reduce noise. Inherent noise from the reflectometry was removed using a Butterworth low-pass or median filter in the spatial domain. Smoothing was also necessary for the subsequent root visualization of rice plants, in which peak finding was used to estimate the locations of root tips. In parallel, notch filters with cutoff frequencies of approximately 1/day were applied to separate the background noise caused by the diurnal temperature effect. Residual transient noise was removed by elementary subtraction of the spatial averages. The background physical strain caused by the spiral structure was compensated for by the elementary subtraction of the temporal averages for the duration before germination, for example, the first 24 h of cultivation.

**Fig. 3:**
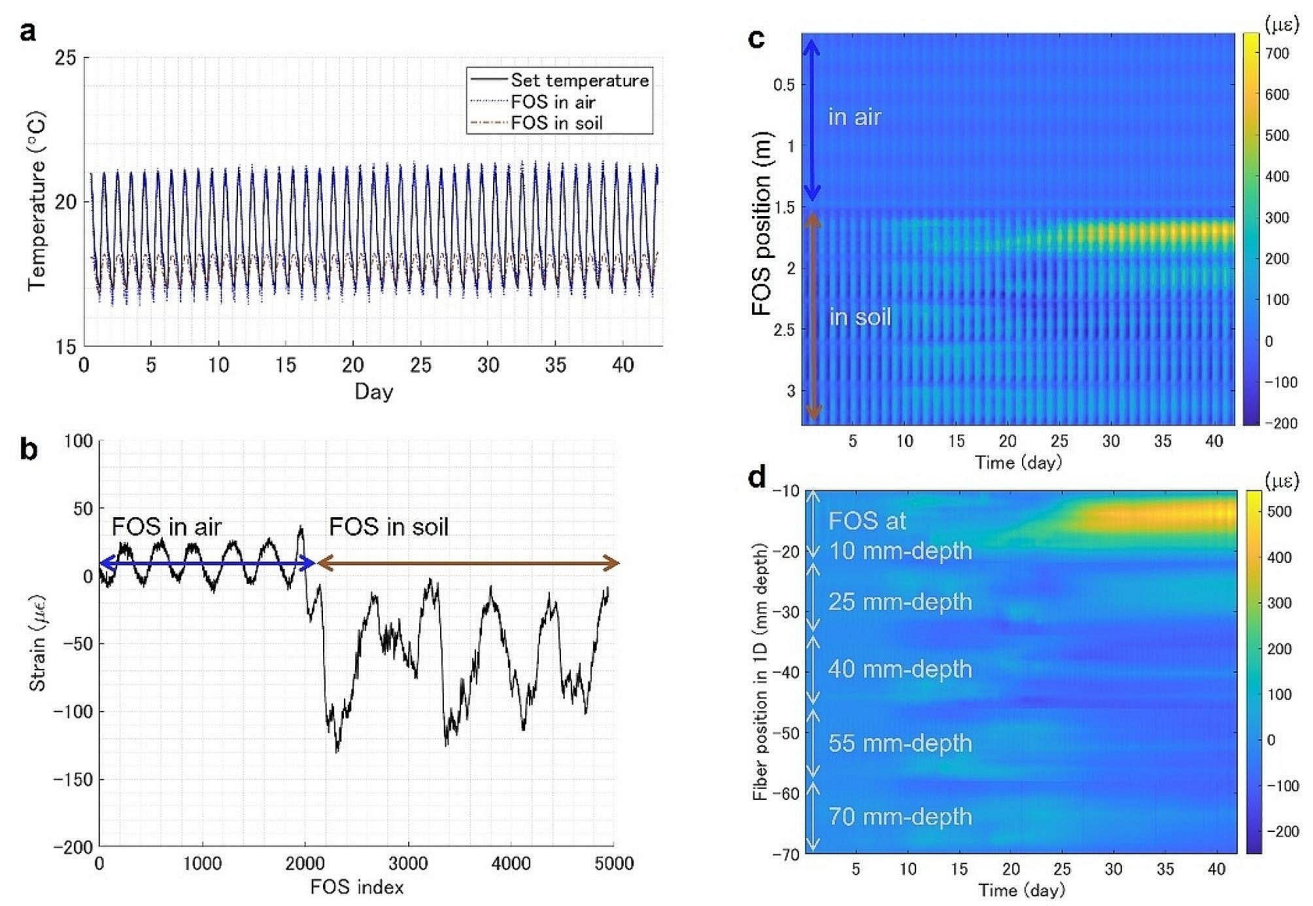
Digital signal processing on a sample data recording radish growth. **(a)** Comparison of time-series spatial averages of FOS placed in the air and the soil and set temperatures of the growth chamber. Raw signals from the FOS in the soil were adjusted for the thermal expansion of PTFE (125 µε °C^− 1^), which is approximately 220 times larger than silica (0.55 µε°C^− 1^). **(b)** The background physical strain indicated by the temporal average along FOS during the pre-germination period. **(c)** Distributed strain recording for the full FOS length before signal processing. **(d)** Distributed strain recording for the underground portion of FOS after signal processing

Figure 3 shows an example of distributed strain recording for root growth in a radish. Notch filters and elementary subtraction of time-series spatial average separated the ambient temperature effects on FOS signals. The subtracted portion of the signals closely followed the temperature of the growth chamber; the spatial average of the FOS placed in the air was nearly identical to the ambient temperature, whereas that of the underground had similar oscillations in frequency but with smaller amplitudes and delays of up to 2.2 h (Fig. 3a). The smaller amplitude and delays underground can be attributed to the insulation and latent heat of the soil.

The structural bias obtained by temporal averaging during the pre-germination period indicated two portions of the FOS: a loose bare fiber reeled in the air and a tightly strained fiber fixed onto the underground device (Fig. 3b).

After signal processing, it shows clearly that local accumulations of strains emerged as the radish root grew (Fig. 3c-d).

### Virtual reconstruction of radish root

To visualize the root development of a radish, we constructed a computational model for tuber crop root growth. Root development has two phases: primary growth occurs as a vertical elongation of the root length, and secondary growth occurs as a lateral expansion of the root radius. The tuber crop root growth is dominated by secondary growth. As Supplementary Theory I describes the details of the secondary root growth model, it assumes root volume expansion causes FOS deformation and estimates the root radius from FOS measurement according to Eq. S1.

**Fig. 4:**
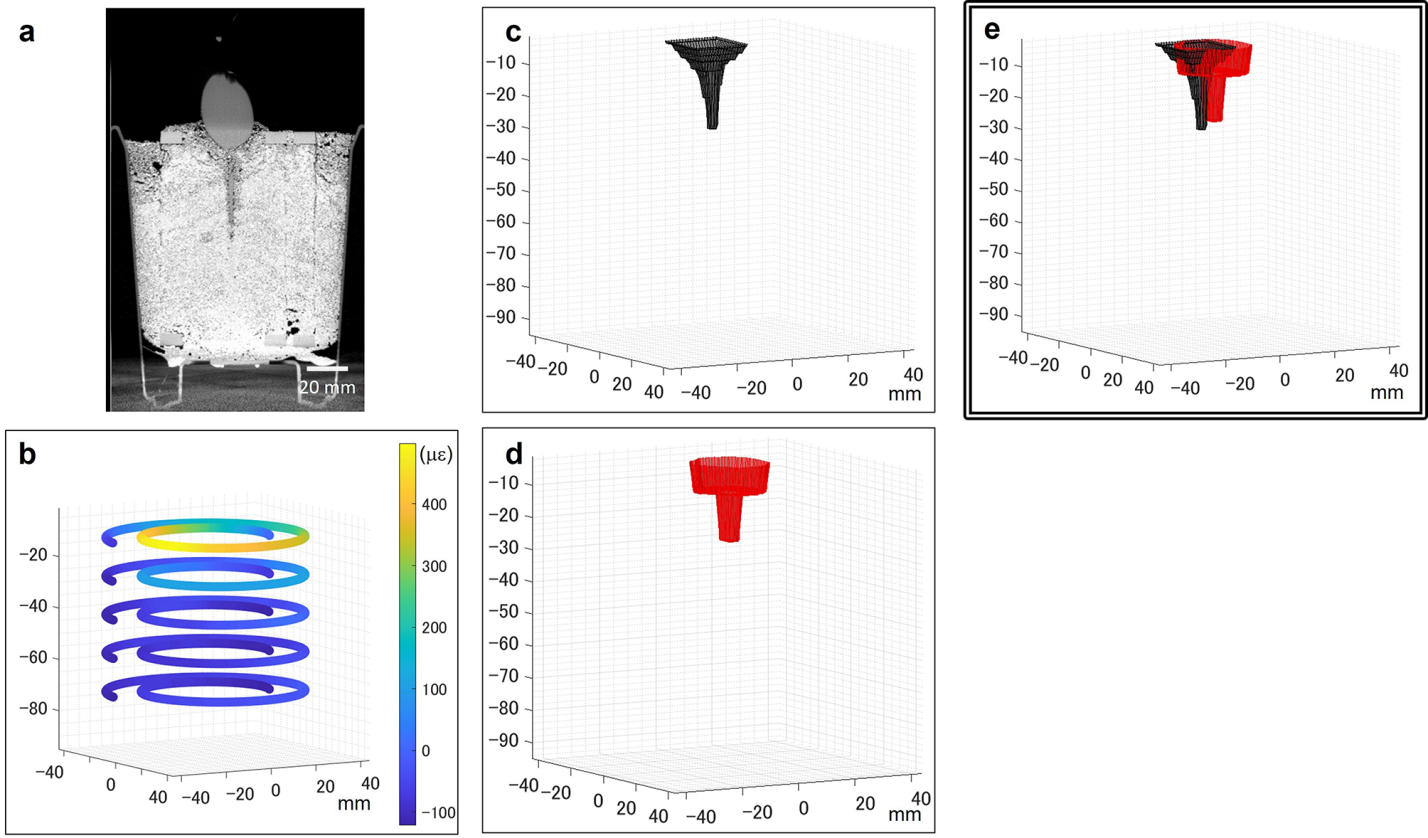
Visualization results from the X-ray CT and FOS measurement on the 42nd day of radish cultivation. **(a)** An image slice from the X-ray CT. **(b)** The spatially encoded heatmap indicating distributed strain and its 3D coordinate from FOS measurement. **(c)** Virtual radish reconstruction from the X-ray CT images by drawing a surface for the root-soil border. **(d)** The virtual radish reconstruction from the FOS measurement, according to Eq. S1. **(e)** Superimposition of panels (c) and (d)

The supplementary movie depicts the time-series distributed strain measurement and virtual reconstruction of the radish root over 42 days after sowing. At the end of the cultivation, an X-ray CT scan and image reconstruction were performed, as shown in Fig. 4. Because the implementation of the FOS limited the vertical resolution to >15 mm, the reconstruction from the distributed strain was coarser than that from the X-ray CT. Nevertheless, the depth of root growth was consistent between the two visualization methods. Dynamic tracking by FOS indicated that the root laterally expanded near the surface on day 12, followed by mild expansion from day 18 to 25, and rapid expansion from day 26 to 37. A local increase of strain indicated root growth at 55 mm depth on day 22, but the root was hardly visible in the X-ray CT images. The radish root excavated at 55 mm depth had a diameter of 0.8 mm (Fig. S4). This suggests that our device can detect a root as fine as 0.4 mm in radius, similar to the standard X-ray CT limit for root detection without image processing [18].

### Virtual reconstruction of rice roots

To visualize the RSA of rice plants, we constructed a computational model for primary root growth. Supplementary Theory II describes the details of the model. Unlike the radish model, the rice model assumes that the downward pressure exerted at the root tip causes FOS strains and estimates the 3D coordinates of the pressure source. The analytical framework can only deduce the distance between the sensor and a root tip. An optimization framework was used to determine the 3D coordinates of root tips. First, peak-finding was used to initialize the coordinates for all root tips. Then the least-squared-error optimization was used to determine the root tip location that matches the array of simulated strains ε_*θθ*_ obtained by Eq. S5 with the array of the actual measurement. Finally, the base of the stem and a root tip were connected by a hyperbolic line to reconstruct an entire root.

**Fig. 5:**
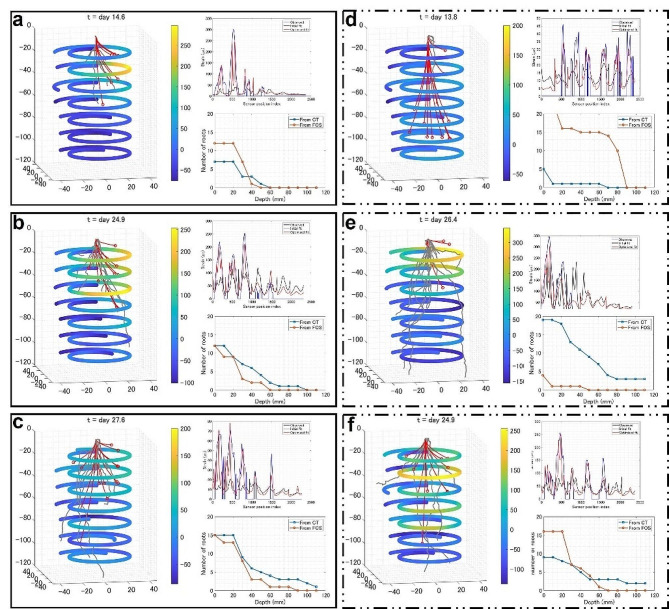
Visualization results from the X-ray CT and FOS measurement of several rice cultivation pots at different growth stages. **(a)** (left) Superimpositions of results obtained on day 15 for Pot #2. Spatially encoded strain heatmap is overlayed with reconstructed rice roots; gray lines indicate CT reconstruction, and red lines indicate FOS reconstructions. (top right) Plots of distributed strain measurement from FOS and simulated strains from the computational model. (bottom right) Plots counting the number of roots along depth for CT and FOS. **(b)** Results for Pot #6 on day 25. **(c)** Results for Pot #7 on day 28. **(d)** Results for Pot #4 on day 14. **(e)** Results for Pot #1 on day 27. **(f)** Results for Pot #4 on day 25

Distributed strain recordings from different rice cultivation pots are shown in Fig. S5. Root growth could be detected as a local strain increment starting from day 10 to 13 after sowing. Virtual rice roots were reconstructed at several time points when cultivation was interrupted to take X-ray CT scans. The two visualization methods at the same time point are compared in Fig. 5. Both methods revealed similar root structures when crown roots grew within the inner layer of the spiral of the FOS device (Fig. 5a-c).

There were some limitations in the FOS reconstruction. For example, the initialization algorithm of peak-finding failed when crown roots were underdeveloped (Fig. 5d). Conversely, when crown roots grew too widely beyond the inner spiral layer, the FOS sensed negative strains due to pressures applied from the opposite side of the PTFE film (Fig. 5e-f). Negative strains were either rejected by thresholding (Fig. 5e) or interfered with positive strains by other root tips that developed inside the inner spiral layer (Fig. 5f), resulting in inaccurate visualization.

## Discussion

The FOS is a promising real-time monitoring technology that is easy to install with a submillimeter longitudinal resolution and is suitable for long-term experiments. We have developed a device that enhances FOS sensing and computational abilities to monitor underground root development. We named the device “Fiber-RADGET” for “Fiber optic sensor-based RADicle gadGET.” The Fiber-RADGET prototype underwent exploration of different materials and orientations for the device backbone. FOS sensitivity was greatly improved by immobilizing it on polymer films such as POM and PTFE. We designed and implemented a spiral concentric backbone that encased the target object to provide spatial encoding for the FOS. The horizontal orientation of the FOS on the PTFE film proved most effective in detecting a thin metal wire that mimicked a crop root. The completed Fiber-RADGET design was able to track root development over weeks and months. Its sensing mechanism relies on detecting local strain increments caused by nearby root growth. Computational models were developed for the root visualization of radish and rice. The virtually reconstructed roots from the FOS generally concorded with the roots traced in high-resolution X-ray CT scans of the actual roots, except in cases of underdeveloped crown roots or roots grown behind the polymer film.

Although radish and rice have two distinct root structures, Fiber-RADGET detected root growth in both as a local strain increment. For the software, we developed two different computational models, each focusing on volume and pressure, to visualize these different root types. Root crops were conveniently visualized, as an enlarged root induces significant deformation in the FOS. However, to extend this method to general dicots beyond root crops, we need to distinguish strains caused by primary and lateral root growth and model each contribution separately for accurate reconstruction.

The accuracy of our pressure-focused primary root growth model was acceptable, despite relying on multiple assumptions. One such assumption is that the force exerted by the root tip is coaxial with gravity, a premise grounded in root gravitropism [39]. Although this applies to most existing cases, crop genetic engineering will likely target the genes responsible for root growth angle to optimize the RSA for water and nutrient uptake [3]. Therefore, the assumption of gravitropism must be reconfirmed in those engineered plants. If necessary, an increased degree of freedom for the pressure direction should be considered in the model, combined with a better heuristic or deep-learning algorithm for more accurate reconstruction.

To date, no high-resolution non-destructive visualization methods have been developed for crop roots in open fields. The Fiber-RADGET has a high potential to be translated to the field like other FOS applications [36–38, 40]. Calibrations of environmental signals affecting FOS measurements, including shrink-swell of soil due to the moisture content, biological activities of worms and moles, and erosion by rainfall, are necessary to isolate the root development signals from compounded measurements. On the other hand, by decoupling signals, Fiber-RADGET can be a multi-throughput environmental monitor that may simultaneously track temperature, soil compaction, and other biological activities in the field. For instance, we demonstrated that the air and soil temperature could be extracted from the background signal in Fig. 3. With appropriate signal processing using frequency filters and cross-correlation, many signals may be isolated and tracked separately to reveal the concealed underground dynamics.

With further adjustments in the device and software, we are confident that Fiber-RADGET enables real-time monitoring of crop roots grown in open fields.

## Methods

### Instrumentation

A high-definition fiber optic strain sensor, HD6S (Luna Inc., Virginia, USA), was used as the FOS., 8-channel ODiSI 6100 reflectometer (Luna Inc., Virginia, USA) was used for high-throughput recording. 0.1–0.2 mm thick NITOFLON (Nitto Denko, Osaka, Japan) and 0.2 mm thick polyacetal (Misumi Group, Tokyo, Japan) sheets were used as polymer films in the prototypes. Films were perforated using a film punch press UDP5000 (Fuji Shoko Machinery, Saitama, Japan) with circular holes 5 or 10 mm in diameter at intervals of 15 mm. The FOS was fixed on the film by 5 mm wide 0.1 mm thick NITOFLON tape (Nitto Denko). The backbone structure was built using polyamide screws (Esco, Osaka, Japan), polycarbonate hexagonal nuts, and 5 mm thick polyacetal plates (Esco). For the completed design, the plates were cut into spiral shapes using a laser cutter Speedy 300 (Trotec, Marchtrenk, Austria). The polymer film was fixed along a pair of spiral plates. The number of turns in the spiral was 1.75 in Figs. 1 and 3, and 4, and 1.5 in Fig. 2, S2, S3. For the direct installation of the FOS in Fig. S1, a PTFE tube with 0.3 mm diameter (Hagitech, Chiba, Japan) was used instead of a film to protect the FOS, and three concentric circles were used to hold the FOS. The structured FOS was placed in an empty pot and filled with materials such as gel and beads (see 4.2 for details). For stress-strain analyses, strains were recorded at either 12.5 or 20 Hz. For plant experiments, strains were recorded twice every 10 min.

### Stress-strain analysis

For abiotic characterization of FOS, a metal wire similar in size to the root was used as a root substitute, according to the prior practices [41, 42]. A stainless wire of 0.5–1.2 mm diameter (Hikari, Osaka, Japan), and the flat side of a fish skewer of 2.5 mm diameter (Izumo Chikuzai Industry, Shimane, Japan) were used as the stress input mimicking the root penetration. For filling, Profile Greens Grade (Profile Product, IL, USA) was used for Fig. S1-S2. The globular glass beads FGB20 (Fuji Manufacturing; Tokyo, Japan) of 0.71–1000 mm diameter were used for Fig. 2. and agarose gel in tris/acetic acid/EDTA (Bio-rad, California, USA) were used for Fig. S3. Force tester MCT1150 (A&D, Tokyo, Japan) was used to measure the force during the wire penetration.

### Digital signal processing

Noises in FOS measurement were removed using the combination of frequency filters and elementary subtractions. The noise reduction based on frequency filtering was obtained by inverting the pointwise product of the Fourier transform of the strain recording and the superposition of a notch filter with the stopband of (duration of the experiment)/ (24 h) and a band-pass filter with the cutoff of (length of the FOS)/ (30 mm). For the background compensation, the temporal average during the pre-germination (first 24 h after sowing) was subtracted for every position and the spatial average of FOS in the device was subtracted for every timepoint of the 2D strain recording.

### Plant materials

The radish (*Raphanus raphanistrum*) line of Kouhaku (Sakata Seed, Kanagawa, Japan) and rice (*Oryza sativa*) line of Kinandang Patong (KP, IRGC#23,364) were used as sample crops in this study. Radish seeds were washed with 70% ethanol for 20 s, rinsed three times with autoclaved water, and stirred in a 2.5% hypochlorite solution for 15 min before being rinsed and incubated with autoclaved water for germination. The KP seeds were immersed in 0.25% Techleed C Flowable (5% ipconazol, 4.6% Cu(OH)_2_; Kumiai Chemical Industry; Tokyo, Japan) at 15 °C for 24 h and in water at 30 °C for two days. Each sterilized seed was sown at the center of the soil at shallow depth. The combination of the FOS and plant materials information (dates of sowing and ending experiments) is given in Table 1.

**Table 1:** Pot number description

Pot #	FOS ID	Start date	End date
1	FS2020LUNA063319	July 1st, 2021	July 28th, 2021
2	FS2020LUNA063328	July 1st, 2021	July 28th, 2021
3	FS2020LUNA063333	July 1st, 2021	July 28th, 2021
4	FS2020LUNA063328	August 6th, 2021	September 10, 2021
5	FS2020LUNA063333	August 6th, 2021	September 10, 2021
6	FS2020LUNA063338	August 6th, 2021	September 10, 2021
7	FS2020LUNA063339	August 6th, 2021	September 10, 2021

### Growth conditions

To use Profile as soil, it was rinsed with tap water three to five times and dried before being filled into four cultivation pots, 70 mm in diameter and 150 mm in height. The profile was saturated pre-cultivation, with a modified Kimura B solution (1.23 mM KNO3, 1 mM KCl, 0.37 mM CaCl_2_, 0.55 mM MgSO_4_, 0.18 mM KH_2_O_4_, and 8.9 µM Fe(II)-EDTA (pH 5.5)) For rice, the saucer was filled with the modified Kimura B solution to a depth of 15 mm. For a radish, the saucer was filled with tap water to a depth of 30 mm. The cultivation was conducted using Biotron (Nippon Medical & Chemical Instruments; Osaka, Japan) with the conditions following the 24 h cycle emulating an average day of Kanagawa in May 2021 (17–21 °C) for radish, and Tsukuba, Japan in July 2017 (25–30 °C, 0-0.5 mmol photosynthetic photon/m^2^/s) for rice. The humidity was set to 50% during the day and 60% at night. The CO_2_ level remained at an average of 400–500 ppm.

### Root visualization

Details about plant root reconstruction from FOS are provided in Supplementary Theory I and II. The model parameters and optimization results for the primary root growth model are listed in Table 2. The custom code for the virtual root reconstruction in MATLAB (MathWorks, Massachusetts, USA) is available at https://github.com/mtei1/Fiber-RADGET.git.

**Table 2:** Parameters used for rice reconstruction

Pot #	Day after sowing	P (GPa)	Initial^1^	Optimal ^2^
1	15	1e5	0.8015	0.6407
2	15	5e5	0.7586	0.5429
3	15	2e5	0.8413	0.6654
1	27	2.5e6	0.865	0.5826
2	27	5e5	0.9734	0.6664
3	27	2e5	0.8812	0.7108
4	25	4e5	0.7483	0.447
5	25	2e5	0.8415	0.6372
6	25	6e5	0.7312	0.4341
7	25	3e5	0.7013	0.5054
4	28	2e5	0.911	0.7106
5	28	1e5	0.8512	0.6555
6	28	2e5	0.9486	0.6375
7	28	1e5	0.817	0.5878

*G* = 0.1 (GPa) and *ν* = 0.5 for all pot numbers

^1^Normalized residual sum of square at initialization

^2^Normalized residual sum of square after optimization

X-ray CT scans were obtained approximately 14 and 28 days after sowing using the X-ray CT system InspeXio SMX-225CT FPD HR (Shimadzu Corporation, Kyoto, Japan), as previously reported [43]. The coordinates for the radish boundaries were manually extracted using Fiji [44], whereas RSAtrace3D in combination with RSAvis3D was used to extract the rice root coordinates [18].

### Electronic supplementary material

Below is the link to the electronic supplementary material.

Supplementary Material 1. This article has accompanying supplementary theories, figures, and a movie file.

## Data Availability

The sample datasets used and analyzed during the current study are available on https://github.com/mtei1/Fiber-RADGET.git or from the corresponding author upon request.
